# Estimating the burden of dementia and parkinsonism through a novel identification algorithm based on healthcare administrative data

**DOI:** 10.3389/fpubh.2025.1622088

**Published:** 2025-10-30

**Authors:** Jacopo Sabbatinelli, Leonardo Biscetti, Marco Lilla, Angelica Giuliani, Francesco Balducci, Deborah Ramini, Giuseppe Rupelli, Marco Pompili, Giuseppe Pelliccioni, Rina Recchioni, Maria Capalbo, Nicola Vanacore, Fabiola Olivieri, Liana Spazzafumo

**Affiliations:** ^1^Department of Clinical and Molecular Sciences, Università Politecnica Delle Marche, Ancona, Italy; ^2^Clinic of Laboratory and Precision Medicine, IRCCS INRCA, Ancona, Italy; ^3^Unit of Neurology, IRCCS INRCA, Ancona, Italy; ^4^Scientific Direction, IRCCS INRCA, Ancona, Italy; ^5^Tech4Care srl, Ancona, Italy; ^6^Regional Health Agency of Marche, Ancona, Italy; ^7^General Direction, IRCCS INRCA, Ancona, Italy; ^8^National Center for Disease Prevention and Health Promotion, Italian National Institute of Health, Rome, Italy

**Keywords:** neurological disorders, dementia, parkinsonism, healthcare administrative databases, prevalence, incidence

## Abstract

**Introduction:**

Neurological disorders (ND), particularly dementia and parkinsonism, are major public health challenges in aging populations. Estimating their prevalence and incidence is essential for healthcare resource planning and targeted interventions. This study aims to estimate the burden of these conditions in the Marche region of Italy, using a novel identification approach applied to administrative healthcare data.

**Methods:**

A cross-sectional study was conducted using administrative databases from the Marche region (2016–2021), including drug prescriptions, hospital discharge records, and chronic condition registries. The TREND protocol was used to enhance case identification. Individuals aged 40 and older were included. Age- and sex-adjusted prevalence and incidence rates were calculated for dementia, parkinsonism, and their co-occurrence. Geographic Information Systems (GIS) were used to analyze spatial distribution.

**Results:**

In 2021, age-adjusted prevalence rates were 7.1‰ for parkinsonism and 31.2‰ for dementia among individuals aged 40 and older, rising to 22.6‰ and 65.8‰, respectively, in those aged 65 and older. Five-year incidence rates were 1.7‰ for parkinsonism and 6.9‰ for dementia. Dementia was more common in women, while parkinsonism predominated in men. GIS revealed higher parkinsonism in southern areas and higher dementia in central and inland areas of Marche. Including antipsychotic and antidepressant prescriptions improved dementia case detection sensitivity.

**Discussion:**

This study demonstrates the value of administrative data and the TREND protocol in improving case identification for neurodegenerative diseases. The observed geographical patterns provide insight for regional healthcare planning in the Marche region. The analysis of antipsychotic and antidepressant use underscores the clinical complexity and healthcare needs of affected individuals. The methodology is scalable and supports reproducible, data-driven strategies for public health policy in aging populations.

## 1 Introduction

Neurodegenerative disorders (NDs) are a major and growing public health challenge due to their high morbidity, mortality, and economic burden. Dementia, the most common consequence of NDs, leads to cognitive impairment and loss of independence, with Alzheimer’s disease (AD) accounting for 60–70% of cases. Patients with dementia experience higher hospitalization rates, prolonged hospital stays, and worse clinical outcomes (1, 2). The progressive increase in life expectancy has contributed to a higher incidence of chronic diseases, including dementia, which are now among the leading causes of disability and mortality (3). With increasing life expectancy, the prevalence of dementia and other chronic diseases continues to rise. By 2050, dementia cases are projected to triple compared to 2019, reaching 153 million worldwide (4). Despite advances in diagnostics and therapeutic management (5, 6), efforts to prevent or delay disease progression remain largely unsuccessful.

Italy, with one of the oldest populations globally, faces a significant burden of age-related diseases. The Marche region, in particular, has a high proportion of older adult residents, with a life expectancy surpassing the national average (7). Dementia accounts for 7.64% of all deaths in Italy, making it the fifth leading cause of mortality (8). Economic costs of dementia in Italy are substantial, with an estimated prevalence of 8.98% among those aged 65 and older, translating to over 1.2 million affected individuals. The associated costs, including informal care, reach approximately €14,000 per patient annually (9).

The increasing prevalence of NDs has led the World Health Organization (WHO) to identify these conditions as a global health priority (8). Healthcare systems must adapt to the growing need for long-term care services and specialized support for aging populations. Accurate, up-to-date estimates of prevalence and incidence are crucial for planning healthcare services. In Italy, however, comprehensive epidemiological data, particularly with long-term and spatial granularity, remain limited (10).

Administrative healthcare databases have emerged as valuable tools for estimating disease burden, tracking care trajectories, and assessing resource distribution (11, 12). However, data fragmentation across different regional sources—including hospital discharge records, drug prescriptions, and medical exemptions—creates challenges in forming a complete epidemiological picture. Integrating these diverse datasets has proven essential for assessing healthcare needs at a population level (13).

This study leverages administrative healthcare data from the Marche region to estimate the prevalence and incidence of dementia and parkinsonism over a 6-year period (2016–2021). By integrating drug reimbursements, hospital admissions, and chronic condition registries, we provide a comprehensive assessment of disease burden. Additionally, we use Geographic Information Systems (GIS) to analyze spatial patterns of disease prevalence and incidence, identifying areas that may require targeted healthcare interventions. The study follows the TREND protocol (Identifying Aging TRajEctories towards chronic Neurodegenerative Diseases), a standardized methodology designed to enhance regional surveillance (14). Supported by the Italian Ministry of Health, this research aims to improve understanding of ND epidemiology and inform data-driven public health strategies.

## 2 Materials and methods

### 2.1 Study design

The TREND Project is a cross-sectional study of permanent residents in the Marche region aged 40 and older. The dataset was constructed by linking drug prescriptions, exemptions, hospital discharge diagnoses, and residential facility data for NDs collected from 2016 to 2021. Patients were categorized into three groups: parkinsonism (Parkinson’s Disease and Atypical Parkinsonism), dementia (AD and non-AD dementia), and coexisting parkinsonism and dementia. Incident cases were defined as individuals with no evidence of neurodegenerative disease in the 5 years prior to diagnosis, across any of the three data sources (hospital discharge records, drug prescriptions, or medical exemptions). A case was considered incident if at least one of these sources indicated a new diagnosis during the study period, with no prior records in any of the others.

### 2.2 Data sources

We utilized four administrative databases: (i) the Regional Population Registry (ARCA) for demographic and administrative data, (ii) the outpatient drug prescription database for National Health Service (NHS)-reimbursed medications, (iii) hospital discharge records (HDRs) containing up to six diagnoses per admission coded using ICD-9, and (iv) exemption records for chronic disease-related co-payment waivers. After matching patient data based on social security numbers, we anonymized the dataset by assigning unique IDs to each patient, ensuring irreversibility and preventing re-identification. This process guaranteed that the original social security numbers could not be traced back from the assigned IDs. To enhance the dataset, we linked it with supplementary databases providing detailed drug information (e.g., commercial name, ATC and AIC codes, and public price) and regional/municipal population data obtained from the Italian National Institute of Statistics (ISTAT). All variables derived from the administrative health records, including demographic information, prescription and hospitalization flags, and exemption codes, are listed in Supplementary Table 1. Georeferencing of data was performed as described in the study protocol (14).

### 2.3 Ethics statement

Ethical approval was not required for the study involving humans in accordance with the local legislation and institutional requirements. No informed consent was required, since as stated in Article 110-bis of the Italian Data Protection Decree, the processing of personal data collected for clinical activities by public and private Scientific Institutes for Research, Hospitalization and Healthcare (IRCCS), for research purposes, does not constitute further processing by third parties, due to the instrumental nature of the healthcare activities carried out by these institutes in relation to research.

### 2.4 Tracer drugs

The list of tracer drugs with ATC codes, exemption codes, and ICD-9-CM diagnostic codes used for case identification is reported in Supplementary Table 2. To identify cases of parkinsonism, we considered levodopa (in its various formulations), and other antiparkinsonian drugs currently used in clinical practice, as reported in the TREND protocol (14).

To identify dementia cases, we included symptomatic drugs for Alzheimer’s disease (AD) and other dementias, such as donepezil, rivastigmine, galantamine, and memantine. Additionally, we considered antipsychotics and the antidepressants mirtazapine and trazodone, excluding patients with psychiatric disorder exemptions or hospital discharge records. This approach, outlined in our protocol (14), is supported by evidence showing that these medications, especially trazodone and mirtazapine, are strong predictors of dementia in older adults (15–17). Antipsychotic drugs, as well as the antidepressants mirtazapine and trazodone, were included among tracer medications to enhance the sensitivity of dementia case identification, minimizing the risk of underestimating dementia cases. These prescriptions contributed to case detection only if no prior evidence of neurodegenerative disease was present in the previous 5 years across any of the three data sources (hospital discharge records, drug prescriptions, or medical exemptions). In this way, prescriptions were considered indicative of incident cases exclusively when aligned with a first-time entry in the administrative history. The list of tracer drugs was reviewed and validated by two board-certified neurologists with expertise in ND (LB and GP).

### 2.5 TREND algorithm

The identification of patients with parkinsonism and dementia followed the TREND algorithm, a standardized protocol developed to enhance case detection using healthcare administrative data. Individuals with parkinsonism were identified based on either (i) repeated prescriptions of antiparkinsonian medications (e.g., ATC codes N04BA, N04BC), or (ii) the presence of a chronic disease exemption code specific for Parkinson’s disease (code 038). Dementia cases were identified if at least one of the following criteria was met: (i) repeated prescriptions of anti-dementia drugs (e.g., ATC N06DA, N06DX01); (ii) hospital discharge records indicating Alzheimer’s disease or other dementia-related diagnoses; (iii) presence of a dementia-related exemption code; or (iv) non-occasional use of antipsychotics, trazodone, or mirtazapine, in the absence of hospitalization or exemptions for psychiatric disorders (e.g., schizophrenia, bipolar disorder, major depression). When only criterion (iv) applied, patients were included only if no evidence of psychiatric illness (diagnosis or exemption) was detected. A full description of the adjudication scheme is reported in Spazzafumo et al. (2024) (14).

### 2.6 Statistical analysis

Between 2016 and 2021, we identified patients diagnosed with Parkinsonism or dementia. For each year, we calculated prevalence and incidence rates based on all entries retrieved. Incident cases were defined as individuals who were newly diagnosed with a neurodegenerative pathology (Parkinsonism or Dementia) during the study period. To be considered an incident case, an individual must not have had a previous diagnosis recorded in our databases in the 5 years prior to the diagnosis date. Each subject was assigned a unique anonymous code to ensure consistent identification across all data sources. Prevalence and Incidence rates were calculated by sex and 5-year age classes (40–44, 45–49, 50–54, 55–59, 60–64, 65–69, 70–74, 75–79, 80–84, 85–89, 90–94, 95–99, and 100 or more). We firstly estimated the prevalence and incidence crude rates of Parkinsonism and Dementia in the Marche region over the period 2016–2021 as the ratio between the number of cases and new cases and the yearly regional population for the considered age range: $r_{t}=\frac{n_{t}}{P_{t}}$ , where *r_t_* is the crude rate, *n_t_* is cases or new cases and *P_t_* is total population in the region for the year *t*. Incidence was expressed as the annual incidence in 2021, calculated as the number of individuals with a first recorded diagnosis in 2021 (after a 5-year disease-free period to exclude prevalent cases) divided by the resident population of the Marche Region in 2021, and reported per 1,000 inhabitants. Mortality rates were estimated with the same approach. For descriptive purposes, annual incidence rates from 2017 to 2020 were also computed; for these years, a correspondingly shorter look-back period was applied.

Age-adjusted estimates of prevalence and incidence were provided based on WHO world standard population for 2000–2025^1^ and the Eurostat standard population^2^ to allow for international comparisons between different age structures: $r_{t}=\sum_{g=1}^{G}\frac{n_{\mathrm{gt}}}{P_{\mathrm{gt}}}\;\omega_{g}$ , where *r_t_* is world population age-adjusted rate, *n_gt_* cases or new cases in age group *g*, *P_gt_* population in age group *g* in the region, *ω_g_* world population share for age group *g*. Ninety-five percent confidence intervals (CIs) were calculated using the Wilson score method for crude and age-specific rates, while CIs for age-adjusted estimates were derived using a bootstrap approach with 5,000 resamplings.

To further assess year-on-year changes, we calculated incidence rate ratios (IRRs) with 95% confidence intervals. IRRs were estimated by comparing crude incidence rates across years. Confidence intervals were computed assuming a Poisson distribution of observed cases.

The spatial analysis is shown at the municipality level. All the data management and analyses were performed using Stata v18.0.

## 3 Results

### 3.1 Incidence and prevalence of parkinsonism and dementia in the Marche region in the year 2021

Using a strict application of the TREND protocol algorithm, we extracted data from the administrative database of the Marche Region, identifying over 5 years (2016–2021) patients aged ≥ 40 years with parkinsonism (group 1) or dementia (group 2) or both dementia and parkinsonism (group 3, which results from the intersection of the group 1 and group 2) (see Supplementary Table 3). Hereafter, we designate 2021 as the reference year, enabling the calculation of the annual incidence rate, defined as the number of new diagnoses in 2021 after applying a 5-year disease-free period (look-back) to exclude prevalent cases. The total population of the Marche Region in 2021 was 1,498,236, including 938,664 individuals aged ≥40 years and 381,162 aged ≥65 years.

**Figure 1:**
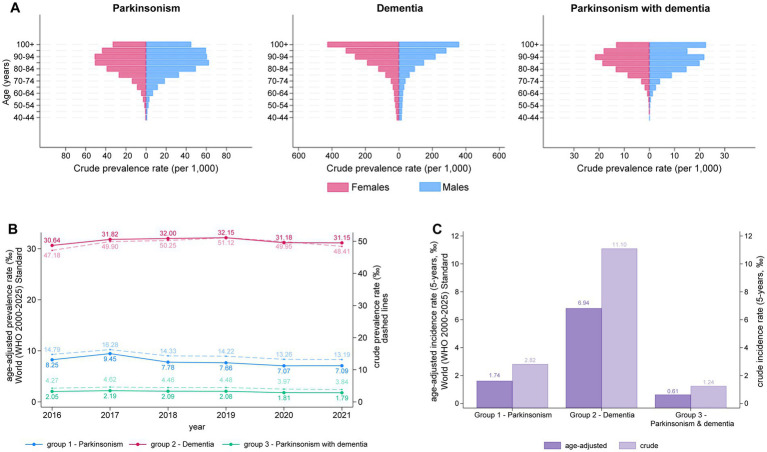
**(A)** Distribution of prevalence of parkinsonism, dementia and parkinsonism with dementia grouped by age (≥40 years) and gender in Marche Region in year 2021. **(B)** Trends in the prevalence of Parkinsonism, Dementia, and Parkinsonism with Dementia from 2016 to 2021. Solid lines represent the age-adjusted prevalence rates (per 1,000) based on the WHO 2000–2025 standard, while dashed lines represent the crude prevalence rates (per 1,000). **(C)** Five-year incidence rates of Parkinsonism, Dementia, and Parkinsonism with Dementia. Dark purple bars represent age-adjusted incidence rates (per 1,000), while light purple bars represent crude incidence rates (per 1,000). The left *y*-axis represents the age-adjusted prevalence/incidence rates, and the right *y*-axis corresponds to the crude prevalence/incidence rate.

We then stratified subjects by age groups with 5-year intervals starting from age 40. Figure 1A displays the crude prevalence rate per 1′000 persons across the three groups. In the parkinsonism group, prevalence rises steadily with age for both males and females, reaching a consistently high level from age 80 onward. In the dementia group, prevalence shows a uniform, continuous increase with advancing age. Lastly, in the third group, prevalence in female peaks in the 90–94 age range, while in males, an additional peak appears among centenarians (Figure 1A). The crude prevalence rate for years prior to 2021 is shown in Supplementary Figure 1.

The sex- and age-specific annual incidence rates and the prevalence for 2021, categorized by age groups, are presented in Table 1. As of December 31, 2021, we estimated that 12,379 individuals with parkinsonism (13.19‰) were living in the Marche region. This corresponds to a prevalence rate of 7.10 per 1,000 inhabitants among individuals aged 40 and older and 22.61‰ among individuals aged 65 and older, after age-adjustment to the WHO World Standard Population. Prevalence increased with age, peaking in the 90–94 age group before declining slightly. The detailed breakdown by sex, available in Supplementary Table 4, shows consistently higher prevalence in men (8.01‰, 95% CI 7.79–8.24) than in women (6.21‰, 95% CI 6.04–6.39).

**Table 1:** Total number of cases, prevalence, annual incidence (new diagnoses in 2021 after a 5-year disease-free period), and annual mortality (all expressed per 1,000 inhabitants) for subjects with parkinsonism, dementia, or parkinsonism with dementia in the year 2021 in the Marche Region of Italy, grouped by 5-year age classes.

Age (years)	Parkinsonism					
	Cases	Prevalence (95% CI)	New cases	Incidence (95% CI)	Deaths	Mortality (95% CI)
40–44	79	0.78 (0.62–0.97)	33	0.32 (0.23–0.46)	2	0.02 (0.01–0.07)
45–49	120	1.02 (0.86–1.22)	50	0.43 (0.32–0.56)	2	0.02 (0.00–0.06)
50–54	242	2.02 (1.78–2.30)	84	0.70 (0.57–0.87)	4	0.03 (0.01–0.09)
55–59	367	3.15 (2.84–3.49)	130	1.12 (0.94–1.32)	4	0.03 (0.01–0.09)
60–64	602	5.87 (5.42–6.35)	184	1.79 (1.55–2.07)	12	0.12 (0.07–0.20)
65–69	934	10.23 (9.60–10.90)	245	2.68 (2.37–3.04)	27	0.30 (0.20–0.43)
70–74	1,443	16.32 (15.51–17.18)	336	3.80 (3.42–4.23)	47	0.53 (0.40–0.71)
75–79	2021	29.87 (28.61–31.17)	463	6.84 (6.25–7.49)	141	2.08 (1.77–2.46)
80–84	2,883	43.78 (42.25–45.37)	566	8.60 (7.92–9.33)	267	4.05 (3.60–4.57)
85–89	2,345	55.43 (53.29–57.65)	353	8.34 (7.52–9.26)	335	7.92 (7.12–8.81)
90–94	1,074	54.15 (51.09–57.39)	167	8.42 (7.24–9.79)	262	13.21 (11.71–14.90)
95–99	250	47.47 (42.05–53.55)	34	6.46 (4.62–9.01)	57	10.82 (8.36–13.99)
100+	19	34.99 (22.51–54.00)	2	3.68 (1.01–13.33)	7	12.89 (6.26–26.37)
Crude	12,379	13.19 (12.96–13.42)	2,647	2.82 (2.71–2.93)	1,167	1.24 (1.17–1.32)
Age-adj (WHO)		7.10 (6.90–7.30)		1.74 (1.66–1.81)		0.46 (0.43–0.49)
Age-adj (Eurostat)		11.48 (11.28–11.68)		2.55 (2.45–2.64)		0.98 (0.92–1.04)
Crude (over 65)	10,969	28.80 (28.27–29.33)	2,166	5.68 (5.45–5.93)	1,143	3.00 (2.83–3.18)
Age-adj (over 65, WHO)		22.61 (22.15–23.04)		4.83 (4.61–5.05)		1.81 (1.69–1.92)
Age-adj (over 65, Eurostat)		26.45 (25.96–26.92)		5.36 (5.14–5.60)		2.54 (2.40–2.69)

Age (years)	Dementia					
	Cases	Prevalence (95% CI)	New cases	Incidence (95% CI)	Deaths	Mortality (95% CI)
40–44	1,529	15.04 (14.31–15.81)	315	3.10 (2.78–3.46)	4	0.04 (0.02–0.10)
45–49	2079	17.75 (17.01–18.52)	423	3.61 (3.28–3.97)	13	0.11 (0.06–0.19)
50–54	2,520	21.08 (20.28–21.91)	509	4.26 (3.90–4.64)	25	0.21 (0.14–0.31)
55–59	2,864	24.58 (23.70–25.48)	612	5.25 (4.85–5.68)	26	0.22 (0.15–0.33)
60–64	2,811	27.39 (26.41–28.41)	614	5.98 (5.53–6.47)	66	0.64 (0.51–0.82)
65–69	3,140	34.39 (33.23–35.60)	730	8.00 (7.44–8.59)	101	1.11 (0.91–1.34)
70–74	3,873	43.81 (42.48–45.18)	929	10.51 (9.86–11.20)	188	2.13 (1.84–2.45)
75–79	4,965	73.37 (71.43–75.36)	1,167	17.25 (16.29–18.25)	332	4.91 (4.41–5.46)
80–84	7,360	111.77 (109.39–114.20)	1825	27.72 (26.49–29.00)	692	10.51 (9.76–11.32)
85–89	7,451	176.11 (172.51–179.77)	1,660	39.24 (37.43–41.13)	1,140	26.94 (25.44–28.53)
90–94	4,970	250.61 (244.62–256.68)	1,182	59.60 (56.39–62.98)	1,189	59.95 (56.73–63.34)
95–99	1,643	311.94 (299.57–324.59)	395	75.00 (68.19–82.42)	512	97.21 (89.50–105.51)
100+	237	436.46 (395.34–478.48)	57	104.97 (81.91–133.59)	82	151.01 (123.35–183.58)
Crude	45,442	48.41 (47.98–48.85)	10,418	11.10 (10.89–11.31)	4,370	4.66 (4.52–4.80)
Age-adj (WHO)		31.15 (30.83–31.49)		6.94 (6.78–7.09)		1.62 (1.57–1.68)
Age-adj (Eurostat)		43.02 (42.64–43.40)		9.81 (9.62–10.00)		3.59 (3.48–3.69)
Crude (over 65)	33,639	88.25 (87.36–89.16)	7,945	20.84 (20.40–21.30)	4,236	11.11 (10.79–11.45)
Age-adj (over 65, WHO)		65.77 (65.02–66.52)		15.55 (15.17–15.93)		6.13 (5.93–6.34)
Age-adj (over 65, Eurostat)		79.65 (78.86–80.44)		18.82 (18.40–19.24)		9.18 (8.91–9.46)

Age (years)	Parkinsonism with dementia					
	Cases	Prevalence (95% CI)	New cases	Incidence (95% CI)	Deaths	Mortality (95% CI)
40–44	5	0.05 (0.02–0.12)	2	0.02 (0.01–0.07)	0	0.00 (0.00–0.04)
45–49	11	0.09 (0.05–0.17)	6	0.05 (0.02–0.11)	0	0.00 (0.00–0.03)
50–54	30	0.25 (0.18–0.36)	11	0.09 (0.05–0.16)	2	0.02 (0.00–0.06)
55–59	50	0.43 (0.33–0.57)	29	0.25 (0.17–0.36)	0	0.00 (0.00–0.03)
60–64	115	1.12 (0.93–1.34)	45	0.44 (0.33–0.59)	3	0.03 (0.01–0.09)
65–69	200	2.19 (1.91–2.52)	74	0.81 (0.65–1.02)	10	0.11 (0.06–0.20)
70–74	327	3.70 (3.32–4.12)	116	1.31 (1.09–1.57)	21	0.24 (0.16–0.36)
75–79	594	8.78 (8.10–9.51)	200	2.96 (2.57–3.39)	56	0.83 (0.64–1.07)
80–84	924	14.03 (13.16–14.96)	304	4.62 (4.13–5.16)	107	1.62 (1.35–1.96)
85–89	817	19.31 (18.04–20.67)	231	5.46 (4.80–6.21)	153	3.62 (3.09–4.24)
90–94	434	21.88 (19.94–24.01)	118	5.95 (4.97–7.12)	107	5.40 (4.47–6.52)
95–99	92	17.47 (14.26–21.37)	24	4.56 (3.06–6.77)	22	4.18 (2.76–6.32)
100+	8	14.73 (7.48–28.80)	1	1.84 (0.33–10.36)	2	3.68 (1.01–13.33)
Crude	3,607	3.81 (3.69–3.94)	1,161	1.23 (1.15–1.30)	483	0.51 (0.47–0.56)
Age-adj (WHO)		1.77 (1.71–1.84)		0.61 (0.57–0.65)		0.18 (0.16–0.20)
Age-adj (Eurostat)		3.23 (3.12–3.33)		1.06 (0.99–1.12)		0.40 (0.37–0.44)
Crude (over 65)	3,396	8.85 (8.56–9.15)	1,068	2.77 (2.61–2.95)	478	1.24 (1.14–1.36)
Age-adj (over 65, WHO)		6.43 (6.20–6.67)		2.10 (1.97–2.24)		0.74 (0.67–0.82)
Age-adj (over 65, Eurostat)		7.99 (7.72–8.26)		2.55 (2.39–2.70)		1.06 (0.97–1.16)

Rates are presented as crude values and age-adjusted estimates based on both the WHO World Standard Population 2000–2025 and the Eurostat 2021 European standard population, all with 95% confidence intervals.

The overall age-adjusted incidence rate of parkinsonism was 1.74 new cases per 1,000 persons in 2021, increasing to 4.83‰ in individuals aged 65 and older. The incidence rate was similar for males and females and rose with age, reaching a peak in the 90–94 age group. Additionally, we estimated an annual mortality rate among individuals with parkinsonism of 0.5‰ (per 1,000 inhabitants) in 2021. Among males, the mortality rate increased steadily with age, whereas in females, it peaked in the 90–94 age range (Table 1).

In the group of subjects with dementia, we identified a total of 45,442 individuals. The age-adjusted prevalence in 2021 was 31.15‰ (30.83–31.49), higher in females (32.30‰, 95% CI 31.85–32.76) than in males (28.85‰, 95% CI 28.36–29.33) and steadily increasing with age. The age-adjusted incidence was 6.94 new cases per 1′000 persons and was higher in females (7.18‰, 95% CI 6.97–7.40) than in males (6.43‰, 95% CI 6.22–6.65), and, like prevalence, consistently increased with age. The mortality rate among individuals with dementia was 1.62‰, with a higher rate observed in males (1.89‰, 95% CI 1.79–1.98) than in females (1.41‰, 95% CI 1.34–1.47) (Supplementary Table 4). The age-adjusted prevalence, incidence, and mortality rates rose to 65.77‰, 15.55‰, and 6.13‰ when considering only individuals aged 65 years and older. Based on the 2021 Eurostat data on the European population structure, the age-adjusted prevalence of dementia was 79.65‰ (Table 1).

In the group of subjects with concomitant parkinsonism and dementia, we extracted a total of 3′607 cases. The age-adjusted prevalence rate was 1.77‰ and was higher in males (1.94‰, 95% CI 1.83–2.04) than in females (1.63‰, 95% CI 1.55–1.71). The age-adjusted incidence rate peaked in 2021 in the 90–94 age group. The annual age-adjusted mortality rate among individuals with parkinsonism and dementia was 0.18‰ (per 1,000 inhabitants) (Table 1).

We reported the age-adjusted prevalence per 1,000 cases for each year from 2016 to 2021, alongside the crude prevalence rate for the population residing in the Marche region. The average prevalence rate, age-adjusted to the World Standard Population, was consistently lower for both parkinsonism and dementia groups compared to the crude prevalence among Marche region residents. Specifically, in 2021, the adjusted prevalence of parkinsonism was 7.09‰ compared to the crude rate of 13.19‰, while the adjusted prevalence of dementia was 31.15‰ compared to the crude rate of 48.41‰ (Figure 1B). A similar pattern was observed for the third group, although the difference between the two rates was less pronounced. Overall, the prevalence of the 3 groups remained stable across the 5-year period (Figure 1B).

We also compared the annual incidence rates for 2021, standardized both to the World Standard Population and to the population of the Marche region. Here too, the crude incidence rate for all 3 groups (i.e., parkinsonism, dementia and parkinsonism + dementia) was higher than the incidence rate standardized to the reference World Population (Figure 1C). Between 2017 and 2021, crude and age-adjusted incidence rates showed an overall decline, with a pronounced reduction in 2020 and a modest rebound in 2021 (Supplementary Figure 2). In particular, incidence of parkinsonism dropped from 3.16 per 1,000 in 2019 to 2.43 in 2020 (IRR 0.77, 95% CI 0.73–0.81), before increasing to 2.82 in 2021 (IRR 1.16, 95% CI 1.10–1.23 vs. 2020). For dementia, the decrease in 2020 was smaller yet significant (6.94 vs. 7.59 per 1,000 in 2019; IRR 0.91, 95% CI 0.88–0.95) and incidence remained virtually unchanged in 2021 (IRR 1.00, 95% CI 0.97–1.04 vs. 2020). Parkinsonism with dementia showed a similar pattern to parkinsonism alone, with incidence falling from 1.49 per 1,000 in 2019 to 1.15 in 2020 (IRR 0.77, 95% CI 0.71–0.84) and only a modest, non-significant increase in 2021 (1.24 per 1,000; IRR 1.08, 95% CI 0.99–1.17 vs. 2020). Estimates for earlier years (2017–2019) should be interpreted with caution because of the shorter look-back window.

### 3.2 Contribution of healthcare record sources

**Figure 2:**
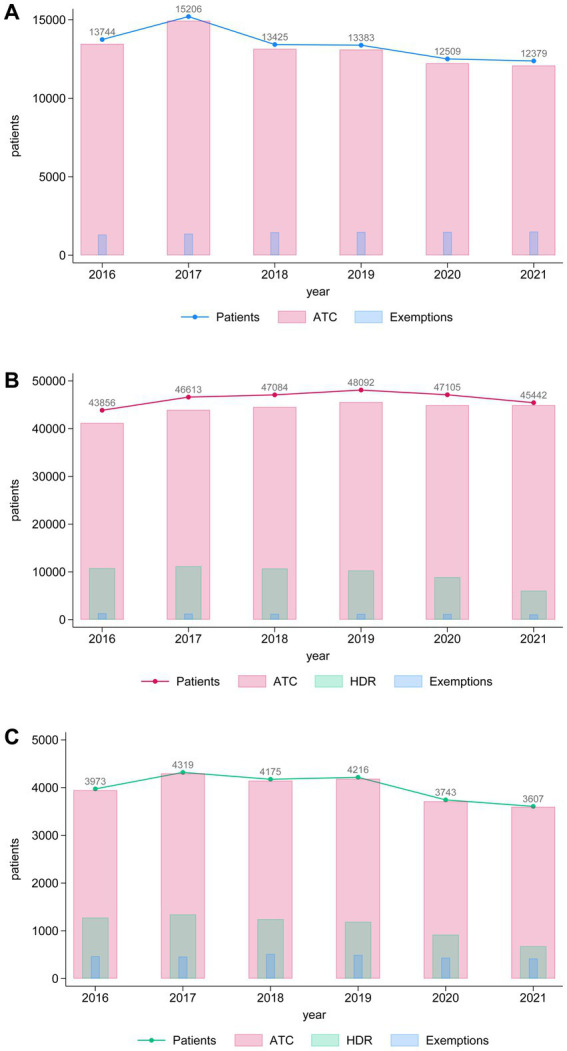
Histograms showing the annual trends in the number of patients (blue line), ATC prescriptions (pink bars), and exemptions (light blue bars) over the years 2016–2021 for **(A)** individuals with parkinsonism, **(B)** dementia and **(C)** parkinsonism concomitant with dementia. The left *y*-axis represents the number of patients, while the bars display the corresponding numbers for ATC prescriptions and exemptions. Numeric values above the line represent the total number of patients per year.

As described in the protocol (14) and in the Materials and Methods section, data extraction was conducted using distinct administrative sources: drug prescriptions (ATC), hospital discharge records (HDR), and service exemptions related to dementia and parkinsonism. As illustrated in Figure 2, most cases for all 3 groups in our study were identified through drug prescription data, with a smaller proportion derived from HDR and exemption records (Figures 2A–C). For groups with subjects with dementia (Figures 2B,C), we observe a higher contribution of HDR compared to the parkinsonism group (Figure 2C). In the third group, data from the HDR effectively captured almost all the identified subjects, with the data from the HDR and exemption records overlapping with the drug prescription data. (Figure 2C). Detailed yearly counts by data source are provided in Supplementary Table 5.

**Figure 3:**
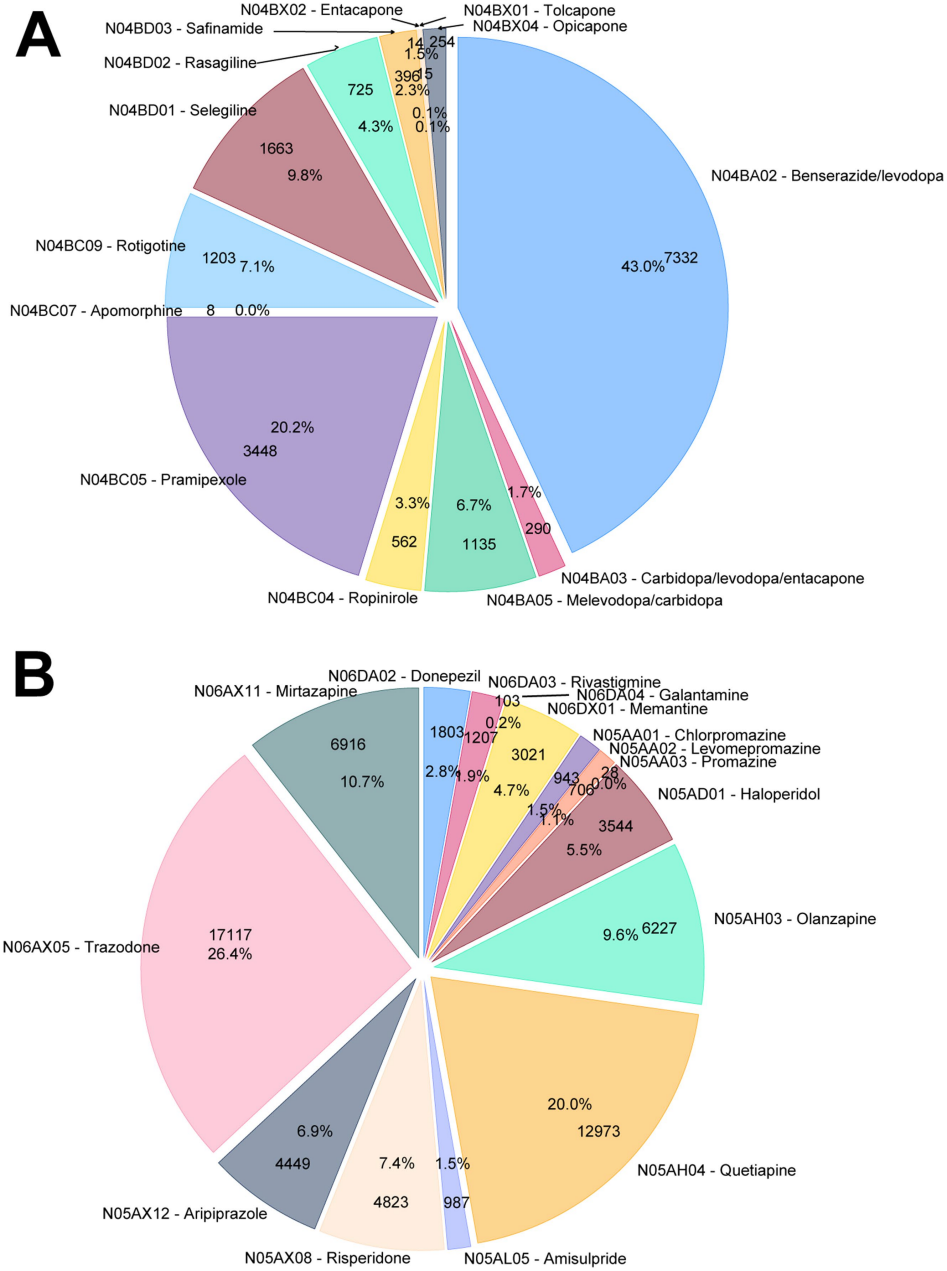
Distribution of the ATC-classified pharmacological treatments for **(A)** parkinsonism and **(B)** dementia.

The pie charts in Figure 3 show the proportions of tracer drugs that contributed to the identification of subjects in the parkinsonism and dementia groups. For the parkinsonism group, 79.5% of the patients were identified as treated with dopa and its derivatives (ATC subgroup N04BA, 49.5%) or other dopamine agonists (ATC subgroup N04BC, 30.0%). For the dementia group, only a small proportion of patients were on anticholinesterase drugs (ATC subgroup N06DA, 4.9%) or memantine (4.7%), while a considerable number of patients were identified due to a prescription of an antipsychotic drug (ATC subgroup N05A, 53.5%) or the antidepressant drugs trazodone (26.4%) and mirtazapine (10.7%).

### 3.3 Georeferencing

**Figure 4:**
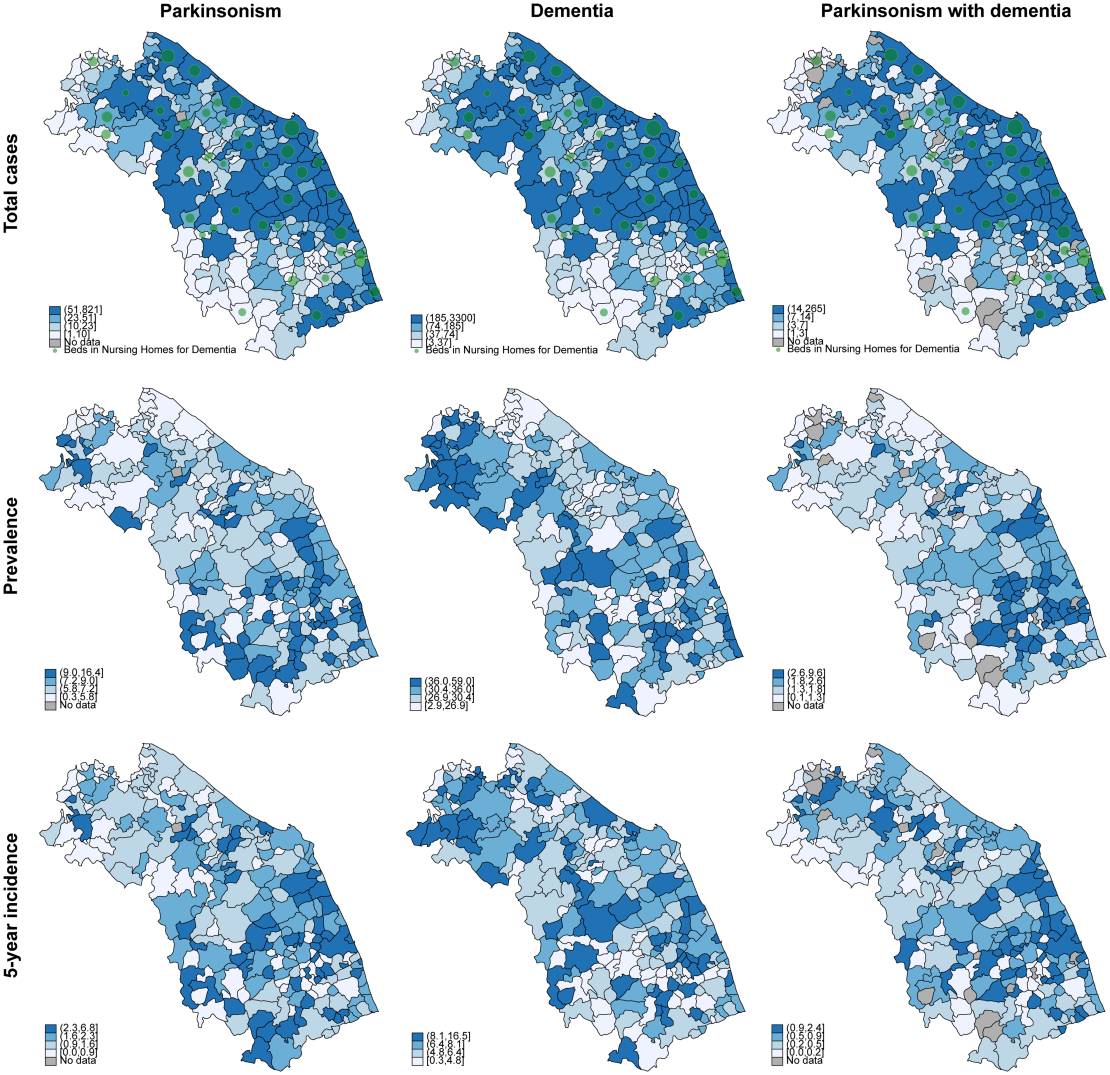
Geographical distribution of total cases, prevalence, and incidence of parkinsonism, dementia, and parkinsonism with dementia in the Marche Region, year 2021. All data are per 1,000 inhabitants. Green circles indicate the availability of beds in nursing homes for dementia care, with larger circles representing a greater number of beds.

Finally, we proceeded with the georeferencing of our data. Geographic Information Systems (GIS) tools enable the analysis and visualization of spatial data by integrating, storing, and manipulating geographically referenced information, producing both interactive and static outputs such as maps. In this case, our goal was to estimate the prevalence and incidence for each municipality in the Marche Region, thus identifying areas with the highest prevalence and incidence of parkinsonism and dementia, and correlating these data with the healthcare services available in the region. Figure 4 presents the absolute number of cases identified across municipalities. A higher number of individuals with dementia and parkinsonism and more nursing home beds for these groups of patients are observed in municipalities with larger populations, which are concentrated in the coastal and northern parts of the region. Figure 4 also shows that the number of hospital beds is lower in inland areas (hilly-mountainous) compared to coastal areas. When analyzing prevalence, we observe some general trends: the prevalence of parkinsonism tends to increase in the southern part of the region, while dementia prevalence shows a more uniformly elevated distribution in the central and more rural areas. Notably, the prevalence of individuals with both parkinsonism and dementia is particularly elevated in the central-southern area (Figure 4). The incidence rate mirrored prevalence distribution on map (Figure 4).

## 4 Discussion

This study aimed to provide an accurate estimation of the prevalence and incidence rates of dementia and parkinsonism in the Marche region of Italy, utilizing three electronic data sources, i.e., drug reimbursement records, hospital admission data, and chronic condition registries.

In summary, among individuals aged over 40 in the Marche region, the adjusted prevalence rate for dementia was 31.2‰. Our findings on dementia prevalence are consistent with existing literature. A 2018 meta-analysis of high-quality observational studies reported an age- and sex-standardized prevalence in people >65 years of dementia in Europe of 7.1% (18), slightly higher than the 6.6% we found. However, it is important to note that different standard populations were used for standardization. In our study, dementia prevalence was standardized using the World Standard Population, whereas the meta-analysis used Eurostat data reflecting the demographic characteristics of the European population. Because the European population is generally older than the World Standard Population, we recalculated our findings using Eurostat data from 2021. This adjustment revealed a prevalence of dementia in the Marche region of 7.97% among individuals aged 65 years and older, slightly higher than the meta-analysis figure but consistent with findings from a similar study conducted in the Campania region of Italy, which reported a prevalence of 7.7% using Eurostat standardization (19). The Campania study employed a methodology similar to ours, utilizing the ICD-9 classification, regional drug prescription records, and exemption databases. This approach was validated against a clinical registry, ensuring its reliability. Like our study, the Campania study used antipsychotic drug prescriptions as a proxy for estimating dementia prevalence and incidence. However, we enhanced this methodology by including two antidepressants commonly prescribed for dementia care, i.e., trazodone and mirtazapine. This likely improved the sensitivity of our protocol in detecting dementia cases.

The slightly higher dementia prevalence observed in the present study compared to the 2018 meta-analysis on dementia prevalence in Europe may be attributed to a potentially higher age-standardized incidence of dementia in Italy in recent years compared to the European Union, as highlighted by a recent study (20). This negative trend in Italy is supposed to be result from modifiable risk factors such as unhealthy diets, low physical activity, and high BMI, which are significant contributors to dementia risk (20).

Regarding incidence, our study found an age-adjusted dementia incidence of 6.94 new cases per 1,000 persons per year among individuals aged >40 years in the Marche region, and 15.2 new cases per 1,000 persons per year in those aged >65 years. These rates slightly exceed those reported in the Italian Longitudinal Study on Aging (ILSA) (21), which found an incidence of 12.47 new cases per 1,000 persons per year among individuals aged 60–84 years. However, the difference is readily explained by methodological variations, as the ILSA study did not include patients aged >85 years. In agreement with the literature (20), our study showed a significantly higher age-standardized prevalence of dementia in women compared to men in Marche region.

The marked reduction in incidence observed in 2020, followed by a modest rebound in 2021, is most likely attributable to the indirect impact of the COVID-19 pandemic, including diagnostic delays, reduced access to healthcare services, and competing mortality. Similar trends have been reported for parkinsonism, dementia, and other neurological disorders during the pandemic, suggesting that part of the apparent decrease may reflect under-detection rather than true changes in disease occurrence (22, 23).

Overall, findings from various studies should be interpreted within the context of an increasing prevalence trend, potentially driven by lifestyle-related factors such as unhealthy diets, low physical activity levels, and elevated BMI, all of which are significant modifiable risk factors for dementia (20).

Among individuals aged over 40 in the Marche region, the adjusted prevalence rates for parkinsonism were 7.1‰. Among individuals aged 65 years and older, the adjusted prevalence rate was 22.6‰. The findings on parkinsonism prevalence in this study are quite consistent with existing literature. For instance, a meta-analysis by Pringsheim et al. (2014) that examined observational studies, including door-to-door surveys and random population samples with physical examinations by healthcare professionals, reported a worldwide prevalence of Parkinson’s disease (PD) of 5.71‰ among individuals aged >40 years (24). The relatively lower prevalence found in Pringsheim meta-analysis compared to our study is likely due to differences in inclusion criteria, as our algorithm is not focused specifically on PD and does not differentiate between PD and atypical parkinsonism.

A 2019 study by Eusebi et al., conducted in the Umbria region of Italy using electronic databases, found a PD prevalence of 5.42‰ among individuals aged >40 years, which is also lower than the 7.1‰ reported in our study (25). Similarly, our reported incidence rate of parkinsonian syndromes (1.7 cases per 1,000 persons per year) was higher than the 0.4 cases per 1,000 persons per year reported by Eusebi et al. This discrepancy can be attributed to differences in inclusion criteria. Unlike Eusebi et al., our analysis did not exclude patients with parkinsonism who were taking antipsychotic medications. Our approach aimed to capture both PD and atypical Parkinsonism cases, regardless of dementia status, rather than focusing exclusively on PD without dementia.

Among patients with parkinsonism, we found a trend of increased prevalence in men compared to women, in agreement with previous investigations (26).

Our study provided valuable insights into the varying rates of dementia and parkinsonism incidence and prevalence across the municipalities of the Marche region. Notably, a geographical pattern emerged: parkinsonism prevalence was higher in the southern part of the region, while dementia prevalence displayed a more uniformly elevated pattern in central and rural areas. In our opinion, these trends are more likely attributable to disparities in access to specialized neurological services for the diagnosis and management of these conditions rather than differences in exposure to risk factors across the region. However, future studies are necessary to explore these differences in greater depth. Specifically, integrating environmental and socioeconomic data, such as pesticide exposure, air quality indices, and education or income levels provided by the Italian National Institute of Statistics (ISTAT), may help to better interpret the observed spatial variations. Such integration would allow disentangling true epidemiological disparities from differences related to diagnostic access or healthcare infrastructure.

Furthermore, with our study, we were able to shed light on the not uniform distribution of health services dedicated to older frail patients in Marche region, with inland areas less covered by targeted services for patients with parkinsonism and dementia. This evidence could represent the basis for specific choice of healthy policy aimed to improve the assistance for these categories of subjects in Marche region. In this regard, health policy actions could include the activation of tele-neurology pathways in underserved inland areas—such as those in the central-southern portion of the Marche region, which show a relatively high burden and limited access to specialized services—and the progressive strengthening of long-term care capacity in rural municipalities, with targets proportionate to disease prevalence. Leveraging the geospatial data presented here may assist in prioritizing resource allocation where gaps are most pronounced.

Overall, our study has several relevant strengths. Indeed, the inclusion of drugs typically used to manage behavioral disturbances in patients with dementia as proxy of dementia condition allowed us to significantly increase our capacity to intercept dementia cases. Indeed, only 10% of dementia patients intercepted by drugs use were individuated by means of typically considered “symptomatic drugs to treat dementia,” i.e., anticholinesterase inhibitors and memantine, while the remaining subjects were identified by considering the use of trazodone, mirtazapine and antipsychotic drugs (mainly quetiapine, olanzapine and risperidone). An Italian validation study conducted on 1,110 patients with dementia and 1,114 control subjects in the community setting, using pharmaceutical prescription (limited to traditional drugs for AD), HDR, residential long-term care records, and exemption data, revealed a sensitivity and specificity of 74.5 and 96.0%, respectively, in identifying cases of dementia (10). It should be noted that cases identified solely through antipsychotic or antidepressant prescriptions lacked confirmatory diagnostic evidence. These drugs were used as proxy indicators of dementia only when no psychiatric diagnoses were present in exemption or hospital discharge records, to reduce misclassification and ensure overall accuracy. However, while the inclusion of these drugs likely enhanced the sensitivity of the proposed algorithm, some loss of specificity is also possible. Therefore, the proposed approach warrants further validation.

Looking ahead, the algorithm is also adaptable to future therapeutic developments. As new disease-modifying drugs for Alzheimer’s disease, such as anti-amyloid therapies, are introduced and assigned ATC codes, they can be incorporated into the tracer drug list for case detection. Over time, the use of such medications may help identify early-stage or prodromal dementia cases more directly, reducing the need for proxy indicators and improving the accuracy of disease burden estimates at the population level.

Furthermore, our data had a very high internal coherence, since there were negligible differences in terms of incidence and prevalence of dementia and parkinsonism across years considered in the study (from 2016 to 2021). Lastly, the protocol developed in this study is highly reproducible across other Italian regions, as the data sources utilized—drug reimbursement records, hospital admission data, and chronic condition registries—are uniformly available nationwide. Given the standardized structure of administrative health data across Italian regions, the protocol is well-suited for multi-regional implementation and comparative validation. Moreover, its ability to produce spatially resolved, age- and sex-stratified indicators lends itself to precision public health approaches, supporting tailored interventions in high-burden populations. Integration of the TREND methodology into routine health monitoring systems may also facilitate policy-relevant insights, enabling more dynamic and data-driven planning at the regional and national levels. Although TREND was implemented within the Italian NHS, its core algorithm is scalable to other settings, as it relies on internationally standardized coding systems (ICD and ATC) for case identification. In countries without universal administrative datasets, equivalent inputs may come from insurance claims, disease registries, or epidemiological programs. Systematic reviews have shown that similar methods can be effectively used to identify dementia and parkinsonism cases using routine health data across diverse healthcare systems (27, 28). Ethical implementation would require adaptation to national data governance regulations, particularly for anonymization and data reuse.

Our study has also some limitations that have to be acknowledged. First, administrative data may contain inaccuracies due to inconsistent coding of neurodegenerative disease diagnoses in health records. Additionally, these data sources cannot capture cases of dementia or parkinsonism in individuals who do not use healthcare services, including those unaware of their condition and therefore undiagnosed. Second, some variability exists in how discharge diagnoses are coded in hospital discharge records (HDRs), despite guidelines requiring the use of the most specific ICD-9 diagnosis codes. However, we expect that most of missed cases are likely to be captured through other sources, i.e., exemption data and/or drugs prescription. Furthermore, in the context of hospitalization, some comorbidities including mild dementia and mild parkinsonism may not be identified in administrative data as these conditions may not have had an impact on the episode of care and as a result would not be coded for in the hospital admission. Considering all these aspects, real dementia and parkinsonism cases in the Marche region are probably higher than those found in our study. Another important limitation of this study is represented by the absence of a correct subclassification of dementia (i.e., AD vs. non-AD dementia) and parkinsonism cases (i.e., PD vs. atypical parkinsonism). To this regard, it is important to keep in mind that a correct distinction could be made only considering ICD-9 codes of HDRs and exemptions records but not using drugs as tracers. In fact, drugs used in AD-related and non-AD dementias are largely the same. For example, acetylcholinesterase inhibitors are prescribed both for Alzheimer’s disease and dementia with Lewy bodies, while antipsychotics are used across different dementia types. Similarly, levodopa is the most commonly used treatment for both Parkinson’s disease and atypical parkinsonism. In this study, we obtained data of prevalence and incidence of parkinsonism and dementia mainly from drug prescriptions and only a minority of cases were identified by ICD codes of HDRs and exemptions records. This hampered the precise subclassification of dementia and parkinsonism cases. In the future, the broader availability of disease-modifying drugs tailored to specific disorders may facilitate a more accurate distinction between different forms of age-related neurodegenerative diseases using healthcare administrative data.

In conclusion, the present study, based on electronic data flows, gives a global estimation of incidence and prevalence of dementia and parkinsonism cases (regardless of subtypes) in Marche region, Italy, roughly as accurate as that furnished by high quality observational studies conducted in other European regions. The present protocol could be easily reproduced in other epidemiological contexts.

## Data Availability

The raw data supporting the conclusions of this article will be made available by the authors, without undue reservation.

## Footnotes

^1^ https://seer.cancer.gov/stdpopulations/world.who.html

^2^ https://ec.europa.eu/eurostat/web/interactive-publications/demography-2025
