# Supplementary material for: Estimating the burden of dementia and parkinsonism through a novel identification algorithm based on healthcare administrative data
Source: Front Public Health. 2025 Oct 30;13:1622088. doi: 10.3389/fpubh.2025.1622088 (PMC12611661; doi:10.3389/fpubh.2025.1622088)
Supplement: Supplementary file 1 [file Data_Sheet_1.pdf]

Supplementary Material

1 Supplementary Figures and Tables

1.1 Supplementary Figures

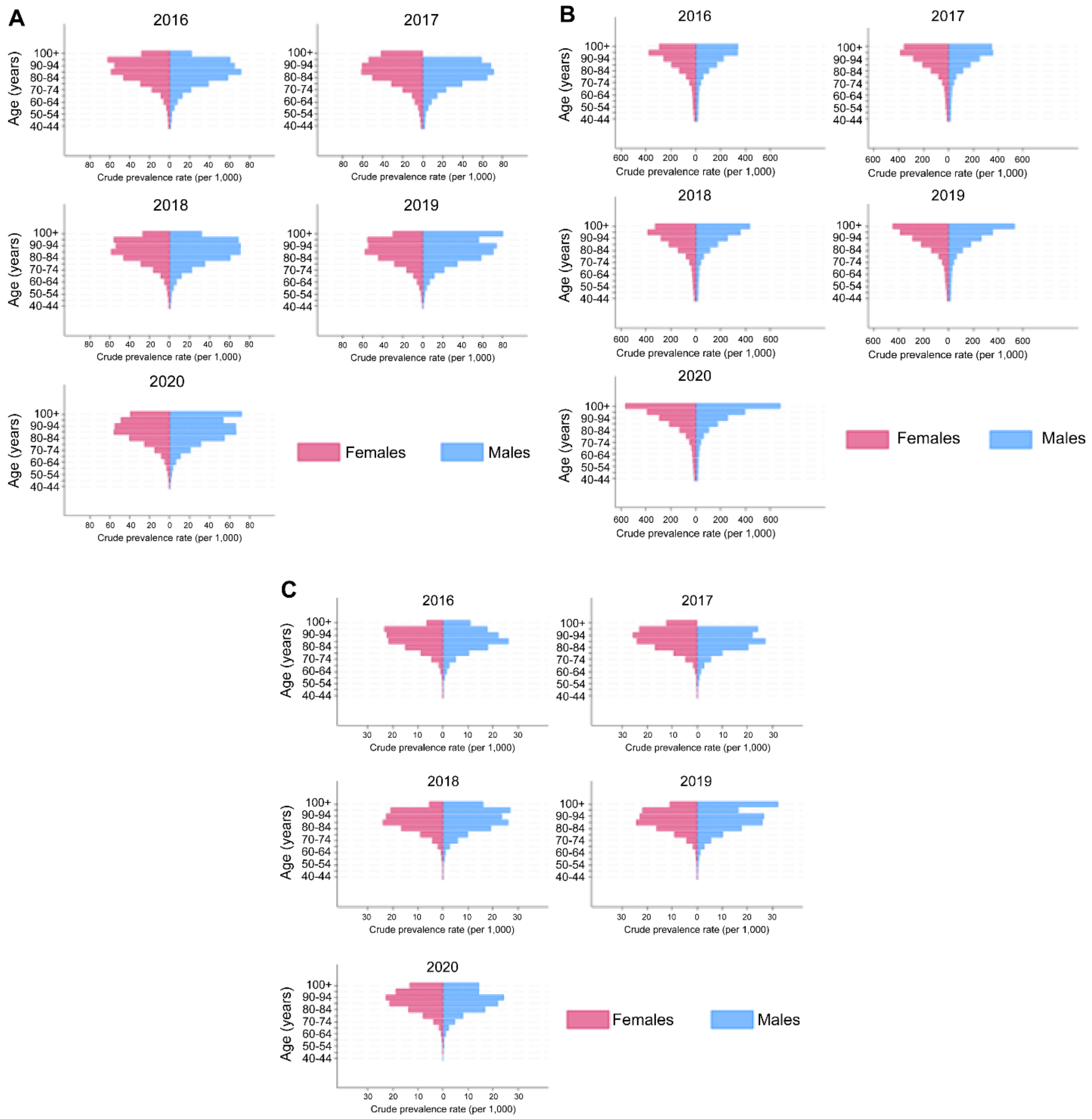

Supplementary figure 1. Distribution of prevalence of (A) parkinsonism, (B) dementia and (C) parkinsonism with dementia grouped by age (≥ 40 years) and gender in Marche Region in years 2016-2020.

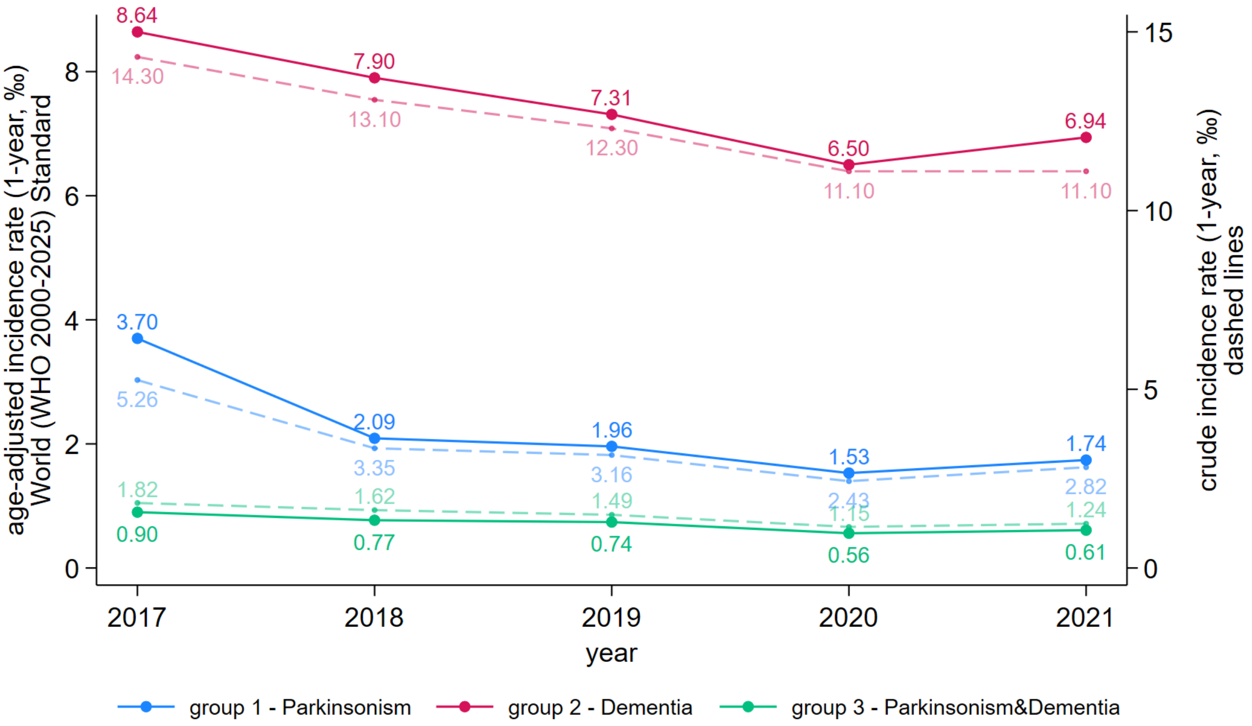

Supplementary figure 2. Trends in the incidence of Parkinsonism, Dementia, and Parkinsonism with Dementia from 2017 to 2021. Solid lines represent the age-adjusted incidence rates (per 1,000) based on the WHO 2000-2025 standard, while dashed lines represent the crude incidence rates (per 1,000). The left y-axis represents the age-adjusted incidence rates, and the right y-axis corresponds to the crude incidence rates.

1.2 Supplementary Tables

Supplementary table 1. List of individual-level variables used in the study, including demographic data, diagnostic and prescription indicators, and geographic identifiers. All variables were derived from routinely collected administrative health records. Binary flags indicate the presence of a condition or event.

Variable Name	Variable Description
pid	Personal Identifier
year	Year
gender	Gender
age	Age
birthdate	Date of Birth
deathdate	Date of Death
citycode	ISTAT Codes the City of Residence
group	Disease group
hdr_g2a	flag for HDR for group 2 Dementia, Alzheimer
hdr_g2b	flag for HDR for group 2 Dementia, Other Dementia
drugs_g2	flag for Drugs for group 2 Dementia
drugs_ps	flag for Drugs for Antipsychotic
exem_ps	flag for Exemption for Psychosis
hdr_ps	flag for HDR for Psychosis
drugs_g1	flag for Drugs for group 1 Parkinsonism
exem_38	flag for Exemption code 38
exem_29	flag for Exemption code 29
exem_11	flag for Exemption code 11

Supplementary table 2. Anatomical Therapeutic Chemical (ATC) codes for tracer drugs, exemption codes, and ICD-9-CM diagnostic codes used for case identification.

Neurodegenerative disease	ATC code of tracer drugs	ICD-9 codes	Exemption codes
Parkinsonism	N04BA02 Benserazide/levodopa<br>N04BA03 Carbidopa/levodopa/entacapone<br>N04BA05 Melevodopa/carbidopa<br>N04BA06 Melevodopa/carbidopa<br>R05DB27 Levodropropizine<br>N04BC04 Ropinirole<br>N04BC05 Pramipexole<br>N04BC07 Apomorphine<br>N04BC09 Rotigotine<br>N04BD01 Selegiline<br>N04BD02 Rasagiline<br>N04BD03 Safinamide<br>N04BX01 Tolcapone<br>N04BX02 Entacapone<br>N04BX04 Opicapone	n/a	038 - Parkinson's disease and other extrapyramidal diseases
Dementia	N06DA02 Donepezil<br>N06DA03 Rivastigmine<br>N06DA04 Galantamine<br>N06DX01 Memantine<br>N05AA01 Chlorpromazine *<br>N05AA02 Levomepromazine *<br>N05AA03 Promazine *<br>N05AD01 Haloperidol *<br>N05AH03 Olanzapine *<br>N05AH04 Quetiapine *<br>N05AL05 Amisulpride *<br>N05AX08 Risperidone *<br>N05AX09 Clotiapine *	331.0 –Alzheimer's disease<br>290 – Dementias<br>290.0 – Uncomplicated senile dementia<br>290.1 – Pre-senile dementia<br>290.10 – Uncomplicated pre-senile dementia<br>290.11 – Pre-senile dementia with delirium<br>290.12 – Pre-senile dementia with delusional aspects<br>290.13 – Pre-senile dementia with depression aspects<br>290.2 – Senile dementia with delusional or depression aspects	029 – Alzheimer's disease<br>011 – Dementia, all subcodes except 011.291.1 (alcohol amnesic syndrome) and 011.294.0 (nonalcoholic amnesic syndrome)

	N05AX12 Aripiprazole *<br>N06AX05 Trazodone *<br>N06AX11 Mirtazapine *	290.20 – Senile dementia with delusional aspects<br>290.21 – Senile dementia with depression aspects<br>290.3 – Senile dementia with delirium<br>290.4 – Vascular dementia<br>290.40 – Uncomplicated vascular dementia<br>290.41 – Vascular dementia with delirium<br>290.42 – Vascular dementia with delusions<br>290.43 – Vascular dementia with behavioral disturbance<br>294.1 – Dementia in other diseases classified elsewhere*<br>294.10 – Dementia in other diseases classified elsewhere, without behavioral disturbances*<br>294.11 – Dementia in other diseases classified elsewhere, with behavioral disturbances*<br>331.1 – Frontotemporal dementia<br>331.11 – Pick's disease<br>331.19 – Other frontotemporal dementias<br>331.2 – Senile degeneration of brain<br>331.3 – Communicating hydrocephalus<br>331.5 – Normal pressure hydrocephalus<br>331.7 – Degeneration of brain in other diseases classified elsewhere<br>331.82 – Dementia with Lewy bodies<br>331.9 – Brain degeneration, unspecified	

* Retained as cases only in the absence of hospitalization for schizophrenia (ICD-9-CM 295.xx), bipolar disorder (296.0x, 296.1x, 296.4x–296.9x), or major depressive disorder (296.2x, 296.3x, 311.xx), and without exemption code ‘044’ (psychoses).

Supplementary table 3. Demographic distribution of cases in the groups Parkinsonism, Dementia, and Parkinsonism with Dementia. The total number of cases for each group is provided, along with the yearly breakdown from 2016 to 2021. The mean age for each group is reported, and the sex distribution is detailed as the number of females, males, and cases where sex data is unavailable (n/a), with corresponding percentages.

	Groups		
	**Parkinsonism**	**Dementia**	**Parkinsonism with dementia**
Total cases for each group	80,646	278,192	24,033
Year			
2016	13,744	43,856	3973
2017	15,206	46,613	4319
2018	13,425	47,084	4175
2019	13,383	48,092	4216
2020	12,509	47,105	3743
2021	12,379	45,442	3607
Mean age (years)	76.7	71.5	80.5
Sex			
Female	42,407 (52.6%)	170,967 (61.5%)	13,449 (56.0%)
Male	37,802 (46.9%)	105,357 (37.9%)	10,472 (43.6%)
n/a	437 (0.5%)	1,868 (0.7%)	112 (0.5%)

Supplementary table 4. Total number of cases, prevalence, annual incidence (new diagnoses in 2021 after a 5-year disease-free period), and annual mortality (all expressed per 1,000 inhabitants) for subjects with parkinsonism, dementia, or parkinsonism with dementia in the year 2021 in the Marche Region of Italy, grouped by 5-year age classes and stratified by sex. Rates are presented as crude values and age-adjusted estimates based on the WHO World Standard Population 2000–2025, all with 95% confidence intervals.

	Females						Males					
	Parkinsonism											
**Age (years)**	**Cases**	**Prevalence (95% CI)**	**New cases**	**Incidence (95% CI)**	**Deaths**	**Mortality (95% CI)**	**Cases**	**Prevalence (95% CI)**	**New cases**	**Incidence (95% CI)**	**Deaths**	**Mortality (95% CI)**
40-44	29	0.57 (0.40-0.82)	14	0.27 (0.16-0.46)	1	0.02 (0.00-0.11)	49	0.97 (0.73-1.28)	18	0.36 (0.22-0.56)	1	0.02 (0.00-0.11)
45-49	46	0.78 (0.59-1.04)	22	0.37 (0.25-0.56)	1	0.02 (0.00-0.10)	71	1.22 (0.97-1.54)	27	0.46 (0.32-0.67)	1	0.02 (0.00-0.10)
50-54	96	1.58 (1.30-1.93)	46	0.76 (0.57-1.01)	1	0.02 (0.00-0.09)	142	2.41 (2.05-2.84)	38	0.65 (0.47-0.89)	2	0.03 (0.01-0.12)
55-59	175	2.97 (2.56-3.44)	65	1.10 (0.86-1.40)	1	0.02 (0.00-0.10)	190	3.30 (2.87-3.81)	64	1.11 (0.87-1.42)	3	0.05 (0.02-0.15)
60-64	260	4.88 (4.32-5.51)	93	1.74 (1.42-2.14)	5	0.09 (0.04-0.22)	337	6.83 (6.14-7.60)	89	1.80 (1.47-2.22)	7	0.14 (0.07-0.29)
65-69	423	8.89 (8.08-9.77)	107	2.25 (1.86-2.72)	4	0.08 (0.03-0.22)	507	11.60 (10.64-12.65)	137	3.14 (2.65-3.70)	23	0.53 (0.35-0.79)
70-74	651	13.95 (12.92-15.05)	174	3.73 (3.21-4.32)	19	0.41 (0.26-0.64)	779	18.67 (17.42-20.01)	158	3.79 (3.24-4.42)	28	0.67 (0.46-0.97)
75-79	1004	27.13 (25.52-28.84)	234	6.32 (5.57-7.18)	60	1.62 (1.26-2.09)	1002	32.68 (30.74-34.72)	221	7.21 (6.32-8.22)	81	2.64 (2.13-3.28)
80-84	1484	39.07 (37.17-41.07)	297	7.82 (6.98-8.76)	106	2.79 (2.31-3.37)	1377	49.42 (46.93-52.02)	264	9.47 (8.40-10.68)	158	5.67 (4.85-6.62)
85-89	1339	50.70 (48.12-53.41)	186	7.04 (6.10-8.13)	165	6.25 (5.37-7.27)	993	62.46 (58.80-66.32)	166	10.44 (8.97-12.14)	169	10.63 (9.15-12.35)
90-94	706	51.07 (47.52-54.87)	91	6.58 (5.37-8.07)	160	11.57 (9.92-13.50)	363	60.42 (54.67-66.73)	74	12.32 (9.82-15.43)	100	16.64 (13.70-20.20)
95-99	178	43.68 (37.82-50.40)	21	5.15 (3.37-7.87)	36	8.83 (6.39-12.21)	71	59.56 (47.49-74.47)	12	10.07 (5.77-17.51)	21	17.62 (11.55-26.78)
100+	15	33.04 (20.12-53.79)	0	0.00 (0.00-8.39)	5	11.01 (4.71-25.52)	4	44.94 (17.61-109.93)	2	22.47 (6.18-78.28)	2	22.47 (6.18-78.28)

Crude	6406	12.89 (12.58-13.21)	1350	2.72 (2.58-2.87)	564	1.13 (1.05-1.23)	5885	13.32 (12.99-13.67)	1270	2.88 (2.72-3.04)	596	1.35 (1.25-1.46)
Age-adj (WHO)		6.21 (6.04-6.39)		1.61 (1.51-1.71)		0.34 (0.31-0.38)		8.01 (7.79-8.24)		1.87 (1.76-1.98)		0.62 (0.57-0.67)
Crude (over65)	5800	27.10 (26.42-27.80)	1110	5.19 (4.89-5.50)	555	2.59 (2.39-2.82)	5096	30.49 (29.68-31.32)	1034	6.19 (5.82-6.57)	582	3.48 (3.21-3.78)
Age-adj (over65, WHO)		20.13 (19.56-20.72)		4.36 (4.07-4.64)		1.34 (1.22-1.48)		25.43 (24.69-26.14)		5.37 (5.03-5.71)		2.45 (2.24-2.66)

Dementia

	**Cases**	**Prevalence (95% CI)**	**New cases**	**Incidence (95% CI)**	**Deaths**	**Mortality (95% CI)**	**Cases**	**Prevalence (95% CI)**	**New cases**	**Incidence (95% CI)**	**Deaths**	**Mortality (95% CI)**
40-44	658	12.91 (11.97-13.93)	157	3.08 (2.64-3.60)	0	0.00 (0.00-0.08)	835	16.48 (15.40-17.62)	141	2.78 (2.36-3.28)	4	0.08 (0.03-0.20)
45-49	995	16.88 (15.87-17.95)	226	3.83 (3.37-4.37)	6	0.10 (0.05-0.22)	1061	18.23 (17.17-19.35)	188	3.23 (2.80-3.73)	6	0.10 (0.05-0.22)
50-54	1289	21.23 (20.12-22.41)	280	4.61 (4.10-5.18)	8	0.13 (0.07-0.26)	1207	20.51 (19.40-21.69)	218	3.70 (3.24-4.23)	16	0.27 (0.17-0.44)
55-59	1566	26.53 (25.27-27.86)	349	5.91 (5.33-6.57)	11	0.19 (0.10-0.33)	1270	22.08 (20.91-23.31)	251	4.36 (3.86-4.94)	14	0.24 (0.15-0.41)
60-64	1557	29.21 (27.82-30.68)	351	6.59 (5.93-7.31)	24	0.45 (0.30-0.67)	1224	24.82 (23.48-26.23)	252	5.11 (4.52-5.78)	41	0.83 (0.61-1.13)
65-69	1723	36.20 (34.56-37.92)	368	7.73 (6.98-8.56)	39	0.82 (0.60-1.12)	1379	31.56 (29.96-33.24)	355	8.12 (7.32-9.01)	62	1.42 (1.11-1.82)
70-74	2224	47.65 (45.75-49.62)	509	10.91 (10.00-11.89)	86	1.84 (1.49-2.27)	1615	38.71 (36.90-40.60)	406	9.73 (8.83-10.72)	102	2.44 (2.01-2.97)
75-79	2977	80.45 (77.72-83.26)	657	17.75 (16.46-19.15)	141	3.81 (3.23-4.49)	1949	63.56 (60.88-66.34)	499	16.27 (14.92-17.75)	190	6.20 (5.38-7.14)
80-84	4666	122.84 (119.58-126.18)	1061	27.93 (26.32-29.64)	349	9.19 (8.28-10.20)	2644	94.89 (91.50-98.38)	740	26.56 (24.73-28.51)	336	12.06 (10.84-13.41)
85-89	5010	189.70 (185.02-194.47)	1013	38.36 (36.11-40.74)	678	25.67 (23.83-27.65)	2388	150.20 (144.73-155.84)	630	39.63 (36.70-42.77)	459	28.87 (26.38-31.59)
90-94	3622	262.01 (254.74-269.40)	785	56.79 (53.05-60.77)	782	56.57 (52.84-60.54)	1314	218.71 (208.44-229.34)	391	65.08 (59.12-71.60)	401	66.74 (60.71-73.34)

95-99	1294	317.55 (303.43-332.01)	296	72.64 (65.07-81.02)	371	91.04 (82.59-100.26)	338	283.56 (258.70-309.81)	95	79.70 (65.64-96.46)	139	116.61 (99.61-136.08)
100+	194	427.31 (382.60-473.24)	44	96.92 (72.99-127.61)	64	140.97 (111.96-176.00)	32	359.55 (267.58-463.15)	7	78.65 (38.62-153.55)	17	191.01 (122.82-284.77)
Crude	27775	55.89 (55.26-56.53)	6096	12.27 (11.96-12.58)	2559	5.15 (4.95-5.35)	17256	39.07 (38.50-39.64)	4173	9.45 (9.17-9.74)	1787	4.05 (3.86-4.24)
Age-adj (WHO)		32.30 (31.85-32.76)		7.18 (6.97-7.40)		1.41 (1.34-1.47)		28.85 (28.36-29.33)		6.43 (6.22-6.65)		1.89 (1.79-1.98)
Crude (over65)	21710	101.44 (100.17-102.73)	4733	22.11 (21.50-22.75)	2510	11.73 (11.28-12.19)	11659	69.76 (68.54-70.99)	3123	18.68 (18.05-19.35)	1706	10.21 (9.74-10.70)
Age-adj (over65, WHO)		70.93 (69.86-71.97)		15.57 (15.04-16.08)		5.44 (5.19-5.68)		57.55 (56.50-58.59)		15.21 (14.66-15.76)		7.08 (6.74-7.44)

Parkinsonism with dementia

	Cases	Prevalence (95% CI)	New cases	Incidence (95% CI)	Deaths	Mortality (95% CI)	Cases	Prevalence (95% CI)	New cases	Incidence (95% CI)	Deaths	Mortality (95% CI)
40-44	0	0.00 (0.00-0.08)	0	0.00 (0.00-0.08)	0	0.00 (0.00-0.08)	5	0.10 (0.04-0.23)	2	0.04 (0.01-0.14)	0	0.00 (0.00-0.08)
45-49	3	0.05 (0.02-0.15)	3	0.05 (0.02-0.15)	0	0.00 (0.00-0.07)	8	0.14 (0.07-0.27)	3	0.05 (0.02-0.15)	0	0.00 (0.00-0.07)
50-54	12	0.20 (0.11-0.35)	8	0.13 (0.07-0.26)	1	0.02 (0.00-0.09)	16	0.31 (0.19-0.48)	3	0.05 (0.02-0.15)	0	0.00 (0.00-0.07)
55-59	21	0.36 (0.23-0.54)	9	0.15 (0.08-0.29)	0	0.00 (0.00-0.07)	29	0.52 (0.37-0.74)	20	0.35 (0.23-0.54)	0	0.00 (0.00-0.07)
60-64	46	0.86 (0.65-1.15)	19	0.36 (0.23-0.56)	1	0.02 (0.00-0.11)	67	1.40 (1.11-1.77)	24	0.49 (0.33-0.72)	2	0.04 (0.01-0.15)
65-69	87	1.83 (1.48-2.25)	34	0.71 (0.51-1.00)	2	0.04 (0.01-0.15)	112	3.34 (2.84-3.93)	40	0.92 (0.67-1.25)	8	0.18 (0.09-0.36)
70-74	150	3.21 (2.74-3.77)	64	1.37 (1.07-1.75)	8	0.17 (0.09-0.34)	175	4.24 (3.66-4.91)	51	1.22 (0.93-1.61)	13	0.31 (0.18-0.53)
75-79	318	8.59 (7.70-9.59)	94	2.54 (2.08-3.11)	27	0.73 (0.50-1.06)	273	9.16 (8.16-10.29)	104	3.39 (2.80-4.11)	29	0.95 (0.66-1.36)
80-84	507	13.35 (12.24-14.55)	165	4.34 (3.73-5.06)	43	1.13 (0.84-1.52)	411	13.78 (12.48-15.22)	135	4.84 (4.09-5.73)	63	2.26 (1.77-2.89)
85-89	493	18.67 (17.10-20.37)	133	5.04 (4.25-5.96)	77	2.92 (2.33-3.64)	317	28.30 (25.84-31.00)	96	6.04 (4.95-7.37)	75	4.72 (3.77-5.91)
90-94	299	21.63 (19.33-24.19)	59	4.27 (3.31-5.50)	64	4.63 (3.63-5.91)	131	31.46 (27.33-36.18)	57	9.49 (7.33-12.27)	41	6.82 (5.03-9.24)
95-99	74	18.16 (14.49-22.74)	19	4.66 (2.99-7.27)	15	3.68 (2.23-6.06)	18	15.10 (9.57-23.74)	5	4.19 (1.79-9.78)	7	5.87 (2.85-12.07)

100+	6	13.22 (6.07-28.53)	0	0.00 (0.00-8.39)	1	2.20 (0.39-12.37)	2	22.47 (6.18-78.28)	1	11.24 (1.99-60.93)	1	11.24 (1.99-60.93)
Crude	2016	4.06 (3.88-4.24)	607	1.22 (1.13-1.32)	239	0.48 (0.42-0.55)	1564	3.54 (3.37-3.72)	541	1.22 (1.13-1.33)	239	0.54 (0.48-0.61)
Age-adj (WHO)		1.63 (1.55-1.71)		0.55 (0.50-0.60)		0.14 (0.12-0.16)		1.94 (1.83-2.04)		0.68 (0.62-0.74)		0.24 (0.21-0.27)
Crude (over65)	1934	9.04 (8.64-9.45)	568	2.65 (2.44-2.88)	237	1.11 (0.98-1.26)	1439	8.61 (8.18-9.06)	489	2.93 (2.68-3.20)	237	1.42 (1.25-1.61)
Age-adj (over65, WHO)		6.07 (5.77-6.37)		1.94 (1.77-2.12)		0.58 (0.50-0.66)		6.87 (6.50-7.24)		2.34 (2.12-2.56)		0.99 (0.85-1.12)

Supplementary table 5. Annual number of individuals identified with parkinsonism, dementia, or parkinsonism with dementia in the Marche Region of Italy (2016–2021), by case-identification criterion (exemption codes, tracer drug prescriptions, and hospital discharge records [HDR]).

			Parkinsonism			
**Year**	**Total cases**	**Exemption 038**		**Drugs**		
**2016**	13744	1316		13468		
**2017**	15206	1376		14939		
**2018**	13425	1464		13148		
**2019**	13383	1484		13104		
**2020**	12509	1486		12230		
**2021**	12379	1507		12086		
			Dementia			
**Year**	**Total cases**	**Exemption 011**	**Exemption 029**	**Drugs**	**HDR (AD)**	**HDR (non-AD)**
**2016**	43856	542	815	41193	1471	3054
**2017**	46613	494	774	43945	1498	3375
**2018**	47084	484	750	44542	1261	3419
**2019**	48092	468	736	45560	1200	3529
**2020**	47105	519	678	44919	914	2802
**2021**	45442	464	617	44927	806	2705
